# Neurofeedback of visual food cue reactivity: a potential avenue to alter incentive sensitization and craving

**DOI:** 10.1007/s11682-016-9558-x

**Published:** 2016-05-27

**Authors:** Niklas Ihssen, Moses O. Sokunbi, Andrew D. Lawrence, Natalia S. Lawrence, David E. J. Linden

**Affiliations:** 1 Cardiff University Brain Research Imaging Centre, School of Psychology, Cardiff University, Cardiff, CF10 3AT UK; 2 Department of Psychology, Durham University, Queen’s Campus, Stockton-on-Tees, TS17 6BH UK; 3 MRC Centre for Neuropsychiatric Genetics and Genomics, School of Medicine, Cardiff University, Cardiff, CF24 4HQ UK; 4 Cognitive Neuroscience Sector, International School for Advanced Studies (SISSA), Trieste, 34136 Italy; 5 Department of Psychology, University of Exeter, Exeter, EX4 4SB UK

**Keywords:** Brain imaging, fMRI, Neurofeedback, Visual cue reactivity, Craving, Food pictures, Obesity, Addiction

## Abstract

FMRI-based neurofeedback transforms functional brain activation in real-time into sensory stimuli that participants can use to self-regulate brain responses, which can aid the modification of mental states and behavior. Emerging evidence supports the clinical utility of neurofeedback-guided up-regulation of hypoactive networks. In contrast, down-regulation of hyperactive neural circuits appears more difficult to achieve. There are conditions though, in which down-regulation would be clinically useful, including dysfunctional motivational states elicited by salient reward cues, such as food or drug craving. In this proof-of-concept study, 10 healthy females (mean age = 21.40 years, mean BMI = 23.53) who had fasted for 4 h underwent a novel ‘motivational neurofeedback’ training in which they learned to down-regulate brain activation during exposure to appetitive food pictures. FMRI feedback was given from individually determined target areas and through decreases/increases in food picture size, thus providing salient motivational consequences in terms of cue approach/avoidance. Our preliminary findings suggest that motivational neurofeedback is associated with functionally specific activation decreases in diverse cortical/subcortical regions, including key motivational areas. There was also preliminary evidence for a reduction of hunger after neurofeedback and an association between down-regulation success and the degree of hunger reduction. Decreasing neural cue responses by motivational neurofeedback may provide a useful extension of existing behavioral methods that aim to modulate cue reactivity. Our pilot findings indicate that reduction of neural cue reactivity is not achieved by top-down regulation but arises in a bottom-up manner, possibly through implicit operant shaping of target area activity.

## Introduction

Visual cues signaling the availability of highly palatable food are omnipresent in the modern media-dominated society and can be potent triggers for approach motivation and ultimately consummatory behavior (e.g. Boswell and Kober 2015; Kemps et al. 2013). Studies examining food cue exposure in individuals with maladaptive eating behaviors, such as obesity, have emphasized the capability of food cues to elicit craving (for a review see Hill 2007). Moreover, while there are important differences between food and drug craving, an emerging perspective highlights strong similarities between neural (and some behavioral) responses to food cues in obesity and to drug cues in addiction (e.g. Berridge 2009; Volkow et al. 2013). According to this view, dopaminergic mesolimbic reward circuits can become sensitized so that food cues acquire incentive salience (Robinson and Berridge 1993) and trigger unconscious ‘wanting’. Consistent with this perspective, brain imaging studies have shown that both obese and drug-addicted patients show heightened activation in response to cues predicting food/drugs (Leyton and Vezina 2013; Stice et al. 2008).

Moreover, neural cue reactivity as indexed by fMRI signals is prospectively associated with food seeking/consumption behavior measured by weight gain (Demos et al. 2012; Yokum et al. 2014) but also predictive of relapse in cocaine (Prisciandaro et al. 2013) and nicotine dependence (Janes et al. 2010). Finally, in both humans and animals the degree of motivational cue reactivity to drug- and food-related stimuli is correlated across domains, suggesting a trait-like susceptibility to externally triggered ‘wanting’ and craving (Stephen V. Mahler and de Wit 2010; Yager and Robinson 2013).

Identifying interventions that can modulate maladaptive brain activation patterns in response to food or drug cues may thus be a promising avenue to treat certain types of overeating or addiction. Most behavioral approaches involving drug/food cues have focused on reappraisal (H. Kober et al. 2010) or cognitive bias modification training (Kemps et al. 2013; R. W. Wiers et al. 2011). One problem associated with altering neural sensitization to reward cues is that cue reactivity related to incentive salience (and predictive of subsequent intake) may primarily be mediated by subcortical structures not easily accessible to conscious control (Robinson and Berridge 1993). For instance, nucleus accumbens responses elicited by pictorial food cues have been shown to predict subsequent snack food consumption irrespective of the level of explicit desire to eat (Lawrence et al. 2012). Moreover, reappraisal of craved foods does not diminish cue-elicited accumbens activation (Giuliani and Mann 2014). Here we present a new fMRI-neurofeedback approach that may facilitate neural desensitization by directly modulating activity levels in the affected motivational networks.

FMRI-based neurofeedback converts Blood Oxygenation-Level dependent (BOLD) signals that are preprocessed and statistically analyzed in real-time into symbolic feedback, which participants can use to self-regulate activation levels in a targeted brain area (Ruiz et al. 2014). Growing evidence suggest that fMRI-neurofeedback training can be used to alter behavioral responses and/or mental states that are associated with the brain areas targeted by neurofeedback. Successful short-term modulation of behavior has been demonstrated in healthy volunteers as well as in some patient populations, e.g. in Parkinson’s disease (Subramanian et al. 2011), stroke (Sitaram et al. 2012) and unipolar depression (Linden et al. 2012), with symptom improvements being measurable for up to two weeks after training (Subramanian et al. 2011). Most clinical neurofeedback studies aimed to *increase* response levels of anatomically or functionally defined regions-of-interest (ROIs) with a known hypoactivation and and/or deficient control function in the targeted condition. In contrast to neurofeedback-guided up-regulation of local BOLD responses, their down-regulation has been considered to be more difficult to achieve (Veit et al. 2012). Moreover, only one group so far has tested whether drug-related/motivational cue responses can be altered by fMRI-neurofeedback (Canterberry et al. 2013; Hanlon et al. 2013; Li et al. 2013). In those studies, nicotine-dependent smokers successfully learned to down-regulate activity of an anatomically defined area (anterior cingulate cortex) during exposure to smoking-related pictures using a thermometer feedback. Neurofeedback was associated with a reduction of craving ratings obtained during the down-regulation run relative to a baseline (no regulation) run. However, in those studies self-regulation runs were always presented *after* baseline runs so that neural adaptation and habituation to the stimuli may have contributed to the observed activation decrease. Further, participants were instructed to ‘allow themselves to crave’ during the baseline run which may have inflated the decrease of craving ratings (and target area activation) in the following self-regulation run. Similar inflation effects may have also contributed to striatal and limbic activation decreases found in smokers who were exposed to smoking cues and instructed to either think about the immediate (positive) feelings (‘now’-strategy, craving-elicitation) or long-term (negative) consequences (‘later’-strategy, craving-reduction) of smoking (H. Kober et al. 2010).

Here we use a novel ‘motivational neurofeedback’ approach that may overcome these limitations and allow for an efficient down-regulation of excessive neural activity and a reliable behavioral modulation even without explicit instruction to crave versus to avoid craving. Instead of presenting BOLD feedback in form of a symbolic indicator in addition to the picture cue (such as the frequently used ‘thermometer’) feedback is given through the cues themselves, with decreasing picture sizes reflecting successful down-regulation and increasing picture sizes reflecting failed down-regulation. This has the advantage that participants do not need to monitor a secondary stimulus for feedback – which may induce dual-task interference. More importantly, regulation is mirrored in real motivational consequences in terms of cue approach (picture growing in size) and avoidance (picture reducing in size). Moreover, our approach includes individually defined target areas based on functional localizer scans taking into account individual differences in motivational neurocircuitry. We have recently reported the feasibility of this approach to achieve target area down-regulation (Sokunbi et al. 2014). Here we show that down-regulation of BOLD responses to food pictures leads to widespread cortical and subcortical neural modulations and that these are potentially associated with experiential and behavioral changes.

## Methods and materials

### Participants

Ten healthy volunteers with no history of eating disorders participated in the study (mean age M = 21.4 years, Standard Deviation (SD) = 2.3). All participants were non-vegetarian females currently taking the combined oral contraceptive pill (to mitigate hormonal effects on food reward processing; Frank et al. 2010). None of the participants were underweight or obese, with their BMI ranging from 20.20 to 28.84 (M = 23.53, SD = 2.66). Participants were comparable in their reported general tendency to exhibit disinhibited eating or experience food cravings as assessed with trait sub-scale of the Modified Trait and State Food Craving Questionnaire (Nijs et al. 2007) before scanning (M = 3.60, SD = 0.79).

Participants were instructed not to eat for at least 4 h before the study and compliance with this procedure was checked verbally before the scan. All participants gave informed consent to participate in the study, which had been approved by the School of Psychology Research Ethics Committee, Cardiff University. Participants were paid £15 for taking part in the study.

### Stimuli and procedure

Neurofeedback training was conducted in a single session and consisted of one functional localizer scan and four runs of motivational neurofeedback training.

#### Functional localizer

Participants were presented with 5 20-s (s) blocks of highly palatable, energy-dense food pictures and 5 blocks of pictures showing neutral household objects in alternating order. Stimuli were sourced from the International Affective Picture System (Lang et al. 2005) and the Internet. Each block contained 5 pictures randomly selected from a total of 20 pictures per category and presented for 2s each. Within each block there were no repetitions of single pictures. The first picture block was preceded by a 22s fixation period; all picture blocks were followed by a 10s fixation period, resulting in a total run length of 222s. Participants were instructed to passively view the pictures, which were back-projected onto a screen behind the MR scanner, through a mirror mounted on the MRI head coil. All pictures were colored and had a 1024 × 768 pixel resolution. Based on the statistical contrast between food and neutral pictures in a whole-brain General Linear Model (GLM) we visually selected for each participant individually a target area showing reliable activation (*t* > 3.0) in the statistical maps derived from the localizer run. Target areas comprised clusters in the amygdala in 5 participants, the putamen/caudate in 2 participants, and the insula, thalamus and parahippocampal gyrus in one participant, respectively (Sokunbi et al. 2014).

#### Motivational neurofeedback

**Fig. 1:**
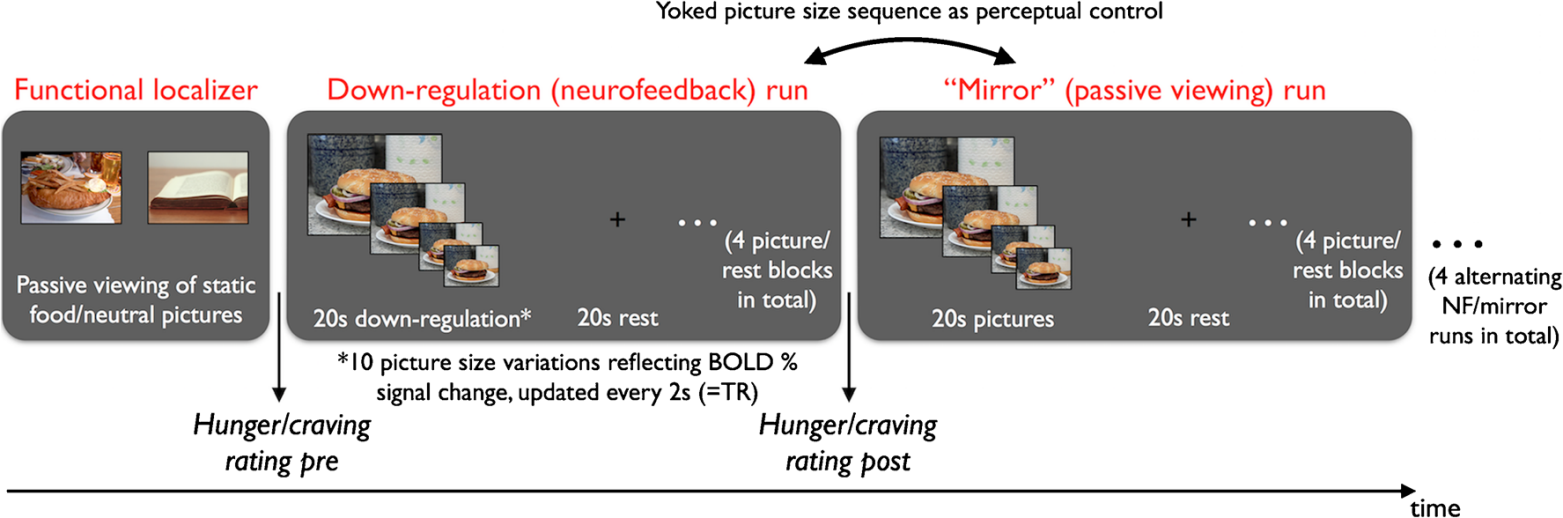
FMRI scanning procedure and task structure of the motivational neurofeedback training with high-caloric food picture cues. The session comprised a functional localizer scan to identify suitable target areas and two down-regulation runs in which feedback about target area activation was given through changes in cue size. Regulation runs were alternated with passive viewing runs (perceptual control/’mirror’ runs)

After the localizer run, participants were presented with four neurofeedback (regulation) runs, each directly followed by a perceptual control (‘mirror’) run showing identical stimuli (see Fig. 1). Each regulation run started with a rest period of 30s showing a fixation cross and was then composed of 4 regulation blocks of 20s duration, which were followed by a rest/fixation period of the same duration. Participants were asked to down-regulate target area activation as long as the pictures were shown. They were informed that the selected target area was involved in food craving/hunger but not given a prescribed strategy to perform the task. In each regulation block a different food image was presented which varied in size depending on the percentage BOLD signal change relative to the preceding fixation block. Using the calibration function described in Sokunbi et al. (2014), images were presented in 10 different possible sizes using a range of 10–100% of the maximum image size (1013 by 760 pixels) and an increment of 10% from one size to the next. The size of the food image was updated every TR (2s) leading to a consecutive display of 10 image sizes of the same picture cue during the 20s-regulation blocks. The exact same size sequence produced by the neurofeedback run was repeated in the subsequent ‘mirror’ run in which participants were asked to passively watch the pictures and which thus provided a perceptual control condition for any BOLD signal changes resulting from the image size variations alone. Across runs 16 different food images were presented showing high-caloric (sweet and savory) dishes (e.g. pizza, burger, chocolate cake). Item-level internal consistency analysis of affective ratings (see below) measured before scanning indicated that the motivational properties of all presented pictures were similar (hedonic valence: Cronbach’s α = 0.80; motivational intensity: Cronbach’s α = 0.79). Because of technical problems, three participants could only complete three instead of four regulation/mirror runs.

#### Behavioral measures

Using the MRI scanner intercom, we asked participants before and after each of the four down-regulation runs to verbally rate their subjective hunger (‘How hungry do you feel?’), satiety (‘How full do you feel?’) and food craving (‘How strong is your desire to eat?’) on a five-point scale that was explained to the participants before the scan. In contrast to the traditional definition of food craving as a desire to eat a specific food (Weingarten and Elston 1990), in the present multi-cue study we measured craving as the general ‘wanting’ of food. This approach is consistent with previous work supporting the validity of the concept of general food craving and highlighting its role in uncontrolled eating, for instance in obesity (Nijs et al. 2007).

In addition to the assessment of immediate behavioral effects of neurofeedback during scanning, we also asked participants to evaluate their (general) food craving before and after the entire training (outside the scanner), using the Modified Trait and State Food Craving Questionnaire (Nijs et al. 2007). Further, we obtained affective ratings of the presented pictures pre and post scanning using three computerized 9-point rating scales relating to specific/categorical hedonic valence of the presented food (‘How pleasant/unpleasant is this specific food [food category]?’) and its motivational intensity (‘Regardless of whether the picture is pleasant or unpleasant, how strong is your reaction to it?’). Finally, food ‘wanting’ was implicitly measured by weighing the amount of crisps (potato chips) taken from a bowl placed next to the participants while filling in a demographic questionnaire after all other assessments had been completed. Participants were simply told that they could eat as much as they want without providing any further information. This behavioral measure of food ‘wanting’ (intake) was previously shown to correlate with food cue-reactivity in the nucleus accumbens but not with subjective hunger (Lawrence et al. 2012).

### MRI data acquisition

Imaging data were acquired using the 3T General Electrics HDx scanner at Cardiff University Brain Research Imaging Centre (CUBRIC). BOLD signals were measured with a T2-weighted gradient echo planar imaging (EPI) sequence that was synchronized to the onset of the task and covered the whole brain. Each volume contained 35 slices of 3-mm thickness, with 1-mm inter-slice spacing (voxel size =3 × 3 × 3 mm, matrix size =64 × 64, TR = 2000 ms, TE = 35 ms, flip angle =80°). High-resolution structural images were acquired after the last functional scan using a fast spoiled gradient echo sequence (FSPGR) with 172 contiguous sagittal slices of 1 mm thickness (voxel size: 1 × 1 × 1 mm, TR 7.9s, TE 3.0 ms, flip angle 20°, FOV 256 × 256 × 172 mm).

### MRI data preprocessing and data analysis

Functional data were preprocessed online using drift removal and 3D motion correction (trilinear interpolation) tools implemented in the Turbo-Brainvoyager™ software. Functional images were realigned and coregistered with participants’ structural scans offline and then spatially normalized to Talairach space. The resulting volume time courses were further preprocessed using spatial smoothing (4 mm Gaussian kernel) and temporal high-pass filtering (2 cycles/time course). Image time series were then analyzed with a whole-brain GLM approach and two regressors modeling onsets/offsets of picture blocks for down-regulation (neurofeedback runs) versus passive viewing (‘mirror’ runs showing identical pictures/size changes). The regressors were convolved with a canonical hemodynamic response function. Beta value estimates for the two regressors were submitted to a second-level, random effect analysis of variance (ANOVA), computing activation maps for the contrast down-regulation - passive viewing. To control for multiple comparisons, we used a cluster-level statistical thresholding approach, which calculates for a given uncorrected *p*-value and volumetric activation map a minimum cluster size necessary to obtain an intended corrected *p*-level using iterative Montecarlo simulations (Forman and Cohen 1995). For the present data, we used an uncorrected *p*-level of *p* < 0.01, based on which the algorithm determined a cluster threshold of 64 voxels (native resolution) to attain a corrected *p*-level of *p* < 0.05.

With regard to the behavioral data, we predicted specific reduction effects in this pilot sample and thus used one-tailed significance tests to compare behavioral measures before and after neurofeedback. Effect sizes for significant differences were calculated using Cohen’s *d* for paired samples (*d* = D / SD_D_, where D is the mean difference score and SD_D_ is the standard deviation of the difference scores). In light of the small sample size we also analyzed behavioral results that showed statistically significant *p*-values with a Bayes approach (Bayes Factors). Bayes Factors allow to robustly quantify the degree of how much the observed data favors the alternative hypothesis (here: reduced hunger/craving after neurofeedback) regardless of sample size (Jarosz and Wiley 2014). For the present data we used the Bayes Factor algorithms implemented in the JASP software (JASP Team 2016), with BF_+0_ based on a directional H1 (pre > post) and a Cauchy prior width of 0.707 (default). We also correlated behavioral measures obtained during scanning with activation levels of individual target areas and limbic clusters identified in the whole-brain analyses. Specifically, we correlated the average difference between ratings of hunger, satiety and craving before and after down-regulation blocks with the difference between the beta weights for the two BOLD signal regressors (regulation and mirror runs) in limbic activation clusters. Beta weight differences were also correlated with the amount of crisps eaten after the scanning. Similar to the results of the simple comparisons between pre- and post-ratings, statistically significant correlations were additionally analyzed by Bayes Factor estimation.

## Results

### Whole-brain effects of target area down-regulation

**Fig. 2:**
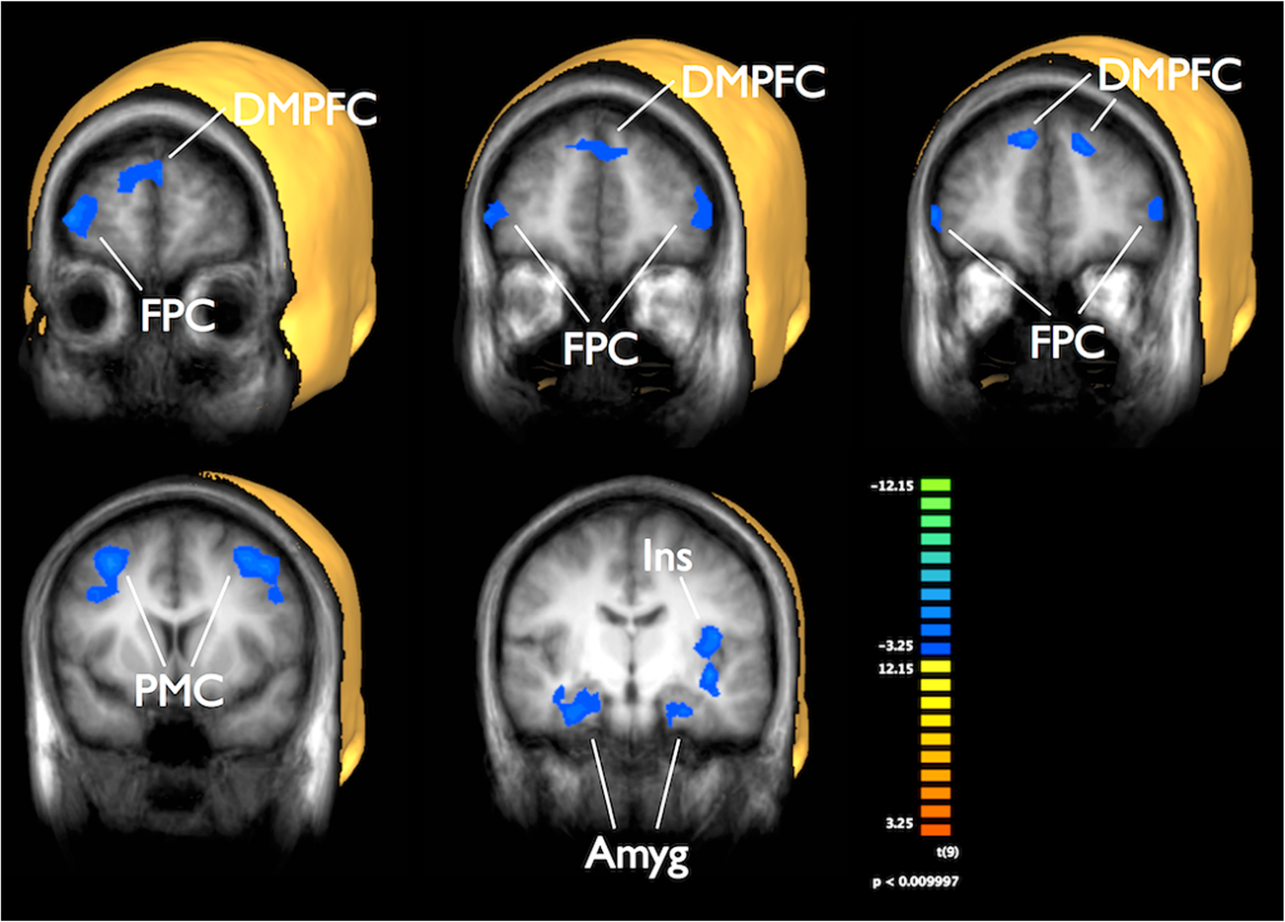
Results of the whole-brain BOLD signal analysis, showing anterior regions with a significant activation difference between down-regulation and mirror runs. Contrast maps illustrate reduced activation during down-regulation in bilateral amygdala (Amyg), left insula (Ins), dorsomedial prefrontal cortex (DMPFC) and frontopolar cortices (FPC). Maps are overlaid on coronal cuts of the averaged, 3d-reconstructed high-resolution structural images

As previously reported, the localizer procedure identified mostly limbic and subcortical areas as having the strongest response to the food cues (amygdala in five participants, basal ganglia areas in two, thalamus in one). The neurofeedback training allowed participants to successfully reduce activation in these individually determined target areas, compared to activation during the mirror runs (Sokunbi et al. 2014). The present results reveal that at the whole-brain level neurofeedback led to functionally specific activation decreases in several cortical and subcortical regions (see Table 1 and Fig. 2). Importantly, this effect included regions with known involvement in food cue reactivity (van der Laan et al. 2011), namely limbic areas (left and right amygdala) and left insula. In addition we found reduced BOLD responses in the dorsomedial prefrontal cortex (DMPFC), frontopolar cortices, premotor cortices, attentional regions (intraparietal sulcus) and higher-order visual areas (precuneus/cuneus and lateral occipitotemporal cortex) as well as in smaller clusters in the middle temporal gyri and postcentral gyri. Inspection of mean beta estimates in the down-regulation condition showed that values were negative for all identified clusters, suggesting that neurofeedback not only reduced the magnitude of activation in response to the cues (compared to the mirror runs) but even led to a de-activation of neural circuits relative to the rest periods. Control analyses of the contrast between down-regulation and baseline (rest) demonstrated that this de-activation excluded primary visual areas, which showed increased BOLD responses during picture presentation ruling out that participants simply closed their eyes to achieve activation decreases. Surprisingly, we did not find that any of the presumed cortical ‘control regions’, such as dorsolateral/ventrolateral prefrontal areas with a known involvement in top-down emotion regulation (H. Kober et al. 2010) showed higher activation in regulation versus passive viewing runs.

**Table 1:** Center of gravity Talairach coordinates and cluster sizes of areas showing reduced activation during down-regulation of cue-elicited BOLD responses relative to passive viewing of the same stimuli

Region (L/R)	Center of gravity (x, y, z)	Cluster size (1x1x1 mm voxels)
Amygdala (L)	−29, −10, −18	1771
Amygdala (R)	27, −13, −17	3055
DMPFC inferior cluster	3, 50, 32	2330
DMPFC posterior cluster (L)	−14, 30, 44	936
DMPFC posterior cluster (R)	15, 32, 44	2092
Frontopolar cortex (L)	−48, 36, 17	2160
Frontopolar cortex (R)	38, 53, 9	2947
Insula (L)	−32, −19, 8	3697
PMC (L)	−36, 12, 44	3814
PMC (R)	32, 12, 40	6543
IPS (L)	−37, −49, 43	10,855
IPS (R)	32, −64, 32	11,061
Lateral occipitotemporal cortex (L)	−38, −70, −10	10,190
Lateral occipitotemporal cortex (R)	42, −62, −12	8458
Precuneus/cuneus	2, −72, 30	7518
Middle temporal gyrus (L)	−63, −38, −8	2135
Middle temporal gyrus (R)	60, −47, −3	925
Postcentral gyrus (L/R)	5, −34, 54	9806
Splenium/posterior cingulate cortex (L/R)	−1, −37, 21	20,228

### Behavioral effects of target area down-regulation

**Fig. 3:**
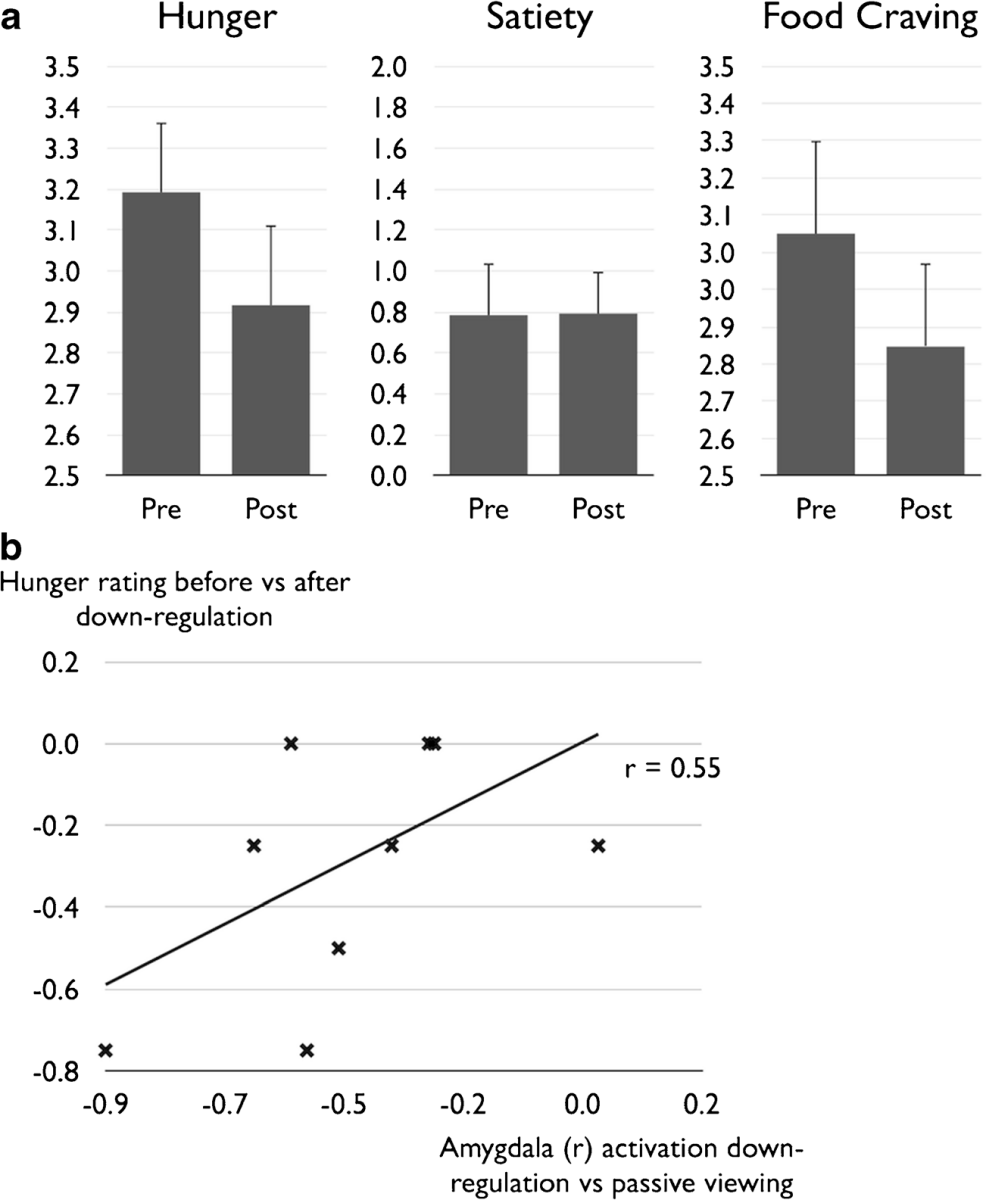
Behavioral effect of motivational neurofeedback during food cue exposure. A Verbal hunger ratings were significantly decreased after down-regulation runs, relative to assessment before down-regulation. Craving ratings showed a similar pattern but the difference missed statistical significance. B The degree of right amygdala activation was positively associated with on-line hunger ratings, with larger activation decreases predicting larger reduction of hunger

Motivational neurofeedback led to a significant reduction of hunger reported after versus before each regulation run (see Fig. 3a), *t*(9) = 2.91, *p* = 0.009, *d* = −0.92. We also found a statistical trend towards the same pattern for ratings of craving, *t*(9) = 1.39, *p* = 0.099, *d* = −0.44. A Bayes factor estimation showed that it was 7.86 times more likely to observe the present hunger rating data under the alternative hypothesis in comparison to the null hypothesis, thus providing substantial evidence for a reduction of hunger after neurofeedback according to canonical interpretations of the Bayes Factor (Jeffreys 1961). For the craving data, Bayes analysis revealed a Bayes Factor of 1.16, suggesting only anecdotal evidence in favor of the hypothesis that cravings were rated lower after neurofeedback. Participants reported no difference between subjective feelings of satiety measured before/after each neurofeedback run, *t*(9) = −0.070, *p* = 0.473.

Importantly, we found preliminary evidence for a significant positive relationship between hunger reduction and down-regulation in the right amygdala cluster identified in the whole-brain analysis, *r* = 0.55, *p* = 0.049 (one-tailed) (see Fig. 3b). Estimation of the Bayes factor suggested that it was 2.75 times more likely for the data to occur under the alternative versus null hypothesis.

We also found positive correlations between activation of left amygdala and hunger reduction, *r* = 0.42, *p* = 0.115, and between activation of the target areas and hunger reduction, *r* = 0.32, *p* = 0.181, which did not reach significance though. Correlations between craving reduction and down-regulation were weak throughout these regions (right amygdala: *r* = 0.12, *p* = 0.37, left amygdala: *r* = 0.07, *p* = 0.42, target area: *r* = −0.24, *p* = 0.26).

In contrast to the above immediate behavioral effects during neurofeedback we did not observe sustained changes of self-reported motivation, as assessed before and after the entire scanning session. Thus, neurofeedback did not result in altered affective ratings (hedonic valence and motivational intensity) of the presented food pictures, all *t*s < 0.77, all *p*s > 0.46, nor did we observe a correlation between amygdala down-regulation and the amount of crisps participants ate when completing the demographic questionnaire, right: *r* = −0.13, *p* = 0.726, left: *r* = 0.24, *p* = 0.497. As expected, trait craving scores did not change across assessment time points, *t*(9) = 1.38, *p* = 0.20. For state craving we found increased scores after the sessions, relative to the pre-experimental assessment, *t*(9) = 4.37, *p* = 0.002. However, homeostatic increase in hunger over time – with the two assessment time points being separated 1.5 h on average – is likely to have caused this effect.

## Discussion

The present findings demonstrate that regional brain activity elicited by cues of high incentive salience (appetitive, high-caloric food pictures after a period of fasting) is reduced during ‘motivational neurofeedback’, in which participants receive real-time feedback of this activity through changes in the cue’s visible size. Motivational neurofeedback allows participants to ‘push’ the cue away from them and thus actively alter its motivational properties in terms of approach versus avoidance (Carver 2006). Our preliminary findings indicate that this picture-size guided neurofeedback may be capable of influencing motivational states, showing a significant reduction of hunger after successful down-regulation. Moreover, the degree of activation reduction in key motivational areas (amygdala) showed some association with the amount of motivational change (hunger reduction) suggesting that behavioral differences were not a mere result of the demand characteristics of the task.

Importantly, we found reduced neural activation during neurofeedback runs relative to passive viewing (‘mirror’) control runs that were physically identical and presented after their corresponding regulation run. Our findings can thus not simply be explained by visual effects related to reduced stimulus size. Moreover, reducing the size of emotional pictures does not affect the magnitude of the Late Positive Potential (De Cesarei and Codispoti 2006), which in turn is correlated with fMRI-based activation measures in motivational regions, such as the ventral striatum measured using fMRI (Sabatinelli et al. 2013).

One may argue that other non-specific factors such as repetitive cue exposure or habituation may have contributed to the reduction of neural activation and hunger. Effects of imagery have been demonstrated when participants were asked to repeatedly imagine eating a specific food, which reduced its subsequent consumption (Morewedge et al. 2010). However, in this study habituation was shown to be stimulus-specific, reducing ‘wanting’/consumption only for the imagined food (chocolate) but not for other food objects (cheese). As single stimuli in our paradigm were not repeated, stimulus-specific habituation was unlikely to have played a major role. Even more importantly, in our critical BOLD contrast we compared the neurofeedback condition with the mirror condition – as the mirror runs were always presented after their corresponding regulation run, any habituation or neural adaptation effect would thus have worked in the opposite direction as our prediction (reduced activation in mirror runs).

Further research is now needed to replicate the successful modification of subjective motivational state by motivational neurofeedback so that, ultimately, our paradigm may complement traditional psychological and pharmacological interventions in controlling maladaptive cue responses, for instance in overeating/obesity. With hardly any behavioral intervention resulting in lasting weight loss in obesity (Turk et al. 2009), there is a clinical need to develop new treatment methods, especially those that target dysfunctional motivational processes, such as cue reactivity and craving. One specific clinical advantage of our paradigm is that it allows to target cue reactivity directly even without the presence of conscious craving. One important question relates to the transferability and stability of neurofeedback-related motivational changes without the presence of feedback. Hunger reduction in the present study occurred only ‘online’ during the scanning session and further studies need to identify tools (e.g. instructed ‘homework’ between multiple training sessions) that facilitate the application of the learned self-regulation skills in a real-life setting, leading to a successful management of craving elicited by environmental cues. The clinical use of fMRI-based neurofeedback may also be facilitated by technologies that allow to map and to feedback neural cue responses in a less costly way, for instance by ‘EEG FingerPrints’ that correlate with local BOLD signals (Meir-Hasson et al. 2014) or by functional near-infrared spectroscopy (S. E. Kober et al. 2014).

On the other hand, the paradigm’s potential clinical utility is indicated by its compatibility with newly developed methods, in which participants learn to overcome their approach bias towards unhealthy food, for instance through implicit re-associations of food cues with avoidance cues (Kemps et al. 2013) or through the use of motivationally opponent response modalities (pulling versus pushing a joystick; Becker et al. 2014).

The present brain imaging results suggest that during motivational neurofeedback motivational networks are de-activated, including limbic areas (amygdala) and left insula. Our results thus emphasize the important role of the amygdala in mediating motivational processes related to cue reactivity. This conforms to brain imaging meta-analyses of food cue reactivity showing that the amygdala is one of the most reliably activated regions during hunger (van der Laan et al. 2011). Moreover, animal work has shown that amygdala-related circuitry plays an important role in incentive motivation for rewards. For example, central amygdala activation can focus incentive salience on food cues (S. V. Mahler and Berridge 2009); whereas the basolateral amygdala triggers reward-seeking behavior through dense, excitatory efferents to the nucleus accumbens suggesting a central influence on motivated actions related to appetitive cues (Stuber et al. 2011).

Our imaging data also show that neurofeedback-guided down-regulation of motivational areas did not require top-down control exerted by prefrontal regions (e.g. dorsolateral and ventrolateral prefrontal cortex), as seen in reappraisal-based regulation of food craving (Giuliani and Mann 2014). Activation of prefrontal control networks, especially VLPFC, is also seen during neurofeedback-guided regulation of emotional areas that involves the use of cognitive strategies, such mental imagery or distancing (Veit et al. 2012). The present modulatory effects during down-regulation may thus be generated by dissociable mechanisms, such as implicit operant learning. Specifically, the reduction of stimulus size may have reinforced activation decreases without the need of inhibitory influences originating from prefrontal areas. We speculate that such successful local ‘shaping’ of target area activation levels was then propagated to other parts of the motivational network in a feed-forward/bottom-up manner. Interestingly, a lack of prefrontal activation increase also characterizes other motivational approaches to re-training cue responses, such as the repeated execution of avoidance movements to alcohol cues (with corresponding decreases in picture size) in alcohol dependence (Ernst et al. 2014; C. E. Wiers et al. 2014).

Finally, another important element of the present approach that may have facilitated effective neural modulation is the individual tailoring of target area selection. In light of the complexity of neural representations of motivation involving networks of multiple brain regions (Richard et al. 2013), identifying those areas that show reliable cue responses at an individual level may provide an adaptive tool to re-train motivational brain circuits.
